# A Synthetic Hydrogel, VitroGel^®^ ORGANOID-3, Improves Immune Cell-Epithelial Interactions in a Tissue Chip Co-Culture Model of Human Gastric Organoids and Dendritic Cells

**DOI:** 10.3389/fphar.2021.707891

**Published:** 2021-09-06

**Authors:** Michelle D. Cherne, Barkan Sidar, T. Andrew Sebrell, Humberto S. Sanchez, Kody Heaton, Francis J. Kassama, Mandi M. Roe, Andrew B. Gentry, Connie B. Chang, Seth T. Walk, Mark Jutila, James N. Wilking, Diane Bimczok

**Affiliations:** ^1^ Department of Microbiology and Cell Biology, Montana State University, Bozeman, MT, United States; ^2^ Chemical and Biological Engineering Department and Center for Biofilm Engineering, Montana State University, Bozeman, MT, United States; ^3^ Department of Chemistry and Biochemistry, Bowdoin College, Brunswick, ME, United States; ^4^ Bozeman GI Clinic, Deaconess Hospital, Bozeman, MT, United States

**Keywords:** microphysiological system, gastric organoid, mononuclear phagocyte, dendritic cell, chemotaxis, matrigel, hydrogel

## Abstract

Immunosurveillance of the gastrointestinal epithelium by mononuclear phagocytes (MNPs) is essential for maintaining gut health. However, studying the complex interplay between the human gastrointestinal epithelium and MNPs such as dendritic cells (DCs) is difficult, since traditional cell culture systems lack complexity, and animal models may not adequately represent human tissues. Microphysiological systems, or tissue chips, are an attractive alternative for these investigations, because they model functional features of specific tissues or organs using microscale culture platforms that recreate physiological tissue microenvironments. However, successful integration of multiple of tissue types on a tissue chip platform to reproduce physiological cell-cell interactions remains a challenge. We previously developed a tissue chip system, the gut organoid flow chip (GOFlowChip), for long term culture of 3-D pluripotent stem cell-derived human intestinal organoids. Here, we optimized the GOFlowChip platform to build a complex microphysiological immune-cell-epithelial cell co-culture model in order to study DC-epithelial interactions in human stomach. We first tested different tubing materials and chip configurations to optimize DC loading onto the GOFlowChip and demonstrated that DC culture on the GOFlowChip for up to 20 h did not impact DC activation status or viability. However, Transwell chemotaxis assays and live confocal imaging revealed that Matrigel, the extracellular matrix (ECM) material commonly used for organoid culture, prevented DC migration towards the organoids and the establishment of direct MNP-epithelial contacts. Therefore, we next evaluated DC chemotaxis through alternative ECM materials including Matrigel-collagen mixtures and synthetic hydrogels. A polysaccharide-based synthetic hydrogel, VitroGel®-ORGANOID-3 (V-ORG-3), enabled significantly increased DC chemotaxis through the matrix, supported organoid survival and growth, and did not significantly alter DC activation or viability. On the GOFlowChip, DCs that were flowed into the chip migrated rapidly through the V-ORG matrix and reached organoids embedded deep within the chip, with increased interactions between DCs and gastric organoids. The successful integration of DCs and V-ORG-3 embedded gastric organoids into the GOFlowChip platform now permits real-time imaging of MNP-epithelial interactions and other investigations of the complex interplay between gastrointestinal MNPs and epithelial cells in their response to pathogens, candidate drugs and mucosal vaccines.

## Introduction

Maintenance of gastrointestinal (GI) homeostasis is essential for proper gut health. Dysregulation of the balance between the GI microbiome, immune system, and epithelium can disturb protective and tolerant responses to commensal and pathogenic bacteria (Kelsall and Rescigno, 2004). Antigen sampling by mononuclear phagocytes (MNPs) including dendritic cells (DCs) and macrophages is essential to maintain this homeostasis (Mann and Li, 2014). MNPs sample their environment, maintaining tolerance to the complex milieu of commensal bacteria and food antigens, while inducing a robust response to pathogens. The mechanism of antigen sampling within the gastric mucosa remains unclear, and investigation of interactions between MNPs and the gastric epithelium are experimentally challenging. We recently demonstrated the uptake of antigen by MNPs through the GI epithelium using co-cultures of human monocyte-derived DCs and *Helicobacter pylori (H. pylori)-*infected gastric spheroids derived from adult gastric tissue (Sebrell et al., 2019). Other groups have successfully co-cultured 3-D gastrointestinal organoids with intraepithelial lymphocytes (Nozaki et al., 2016), T cells (Dijkstra et al., 2018), and both DCs and cytotoxic T cells (Chakrabarti et al., 2018). Sophisticated microphysiological systems (MPSs) that integrate gastrointestinal epithelial cell monolayers and immune components also have been established by multiple groups (Kim and Ingber, 2013; Kim et al., 2016; Shin and Kim, 2018; Beaurivage et al., 2020). However, monolayer systems frequently lack critical features of 3-D spherical organoids, such as oxygen gradients, crypt and gland formation and physiological tissue patterning (Williamson et al., 2018). To date, few studies have developed MPS that utilize organoids in their 3-D configuration, and these systems have yet to introduce immune cell components (Lee et al., 2018; Sidar et al., 2019).

While organoids are ideal models for functional studies of primary epithelial cells, the co-culture of leukocytes with 3-D organoids proves problematic, particularly due to the extracellular matrix (ECM) needed for organoid maintenance. The ECM chosen for co-culture must support the spherical, 3-D organoid structure while still allowing for leukocyte motility and chemotaxis. Cellular motility through 3-D matrices is less understood than motility in two dimensions, as cells are more constrained by their specific environment. ECM constraints can include concentration of adherence factors, matrix elasticity, pore size, and diffusion and adherence of chemotactic agents (Gilat et al., 1994; Zaman et al., 2006; Miron-Mendoza et al., 2010). The constraining factors of matrices are considered in this study, as we identify matrices optimal for MNP and human gastric organoid (HGO) co-culture by comparing MNP chemotaxis through Matrigel, a gelatinous basement membrane mixture derived from mouse sarcoma cells that is used frequently for organoid culture, and chemotaxis through synthetic ECMs. The microphysiological organoid system established in this study, to our knowledge, is the first of its kind to integrate human DCs with gastric epithelium, in the form of 3-dimensional HGOs, on a millifluidic chip platform. Using this system, immune cell-epithelial interactions can be studied with real-time imaging and sampling.

## Materials and Methods

### Tissue Chip Design and Fabrication

**FIGURE 1:**
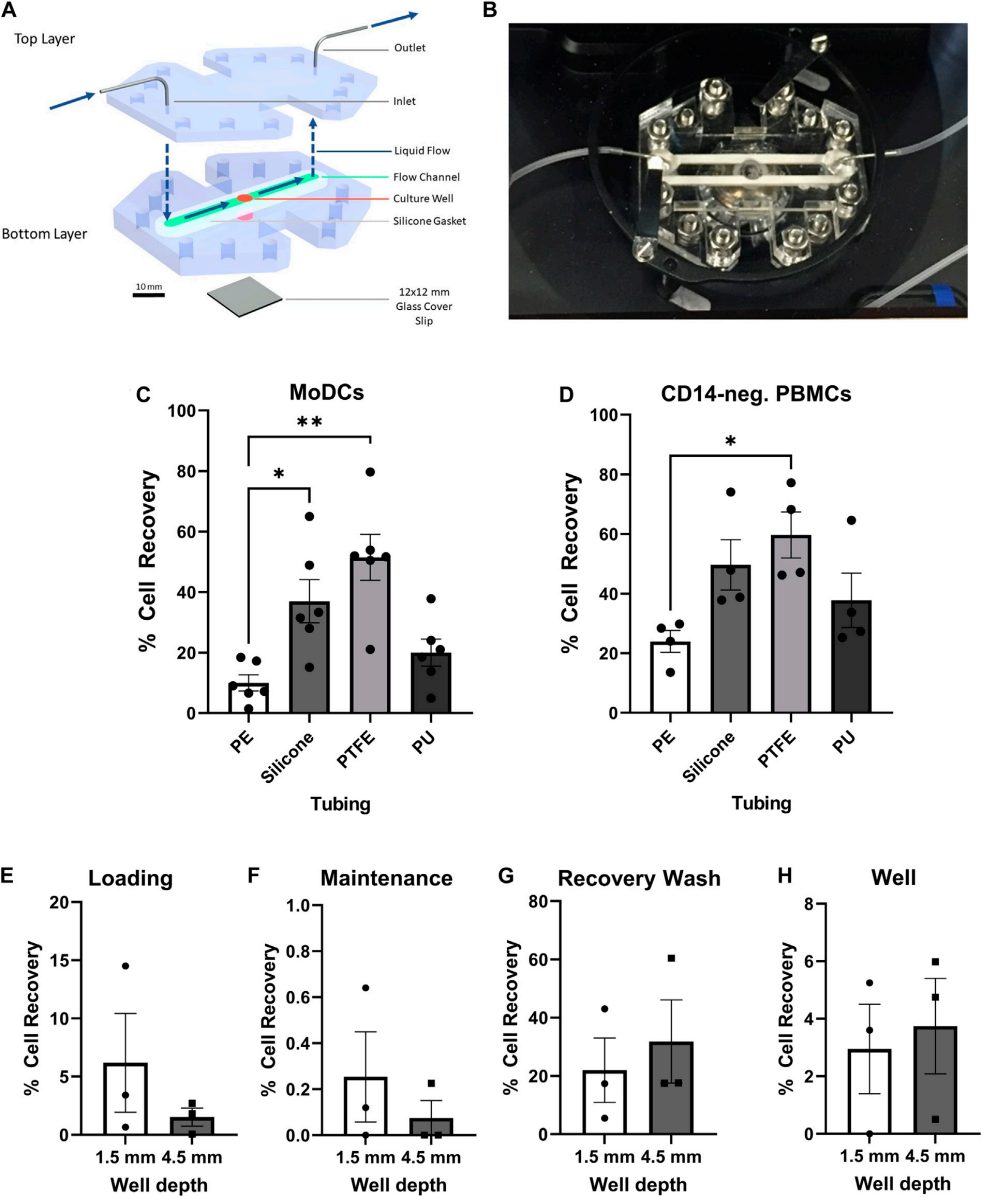
Optimization of the GOFlowChip for integration of dendritic cells. **(A)** Schematic representation of the GOFlowChip, depicting the two-layer PMMA body (blue), central culture well (red), silicone gasket (green), and glass cover slip bottom (gray). Blue arrows indicate direction of liquid flow. **(B)** Photographic image of a GOFlowChip. Recovery of **(C)** MoDCs and **(D)** CD14^–^ PBMCs after flowing cell suspensions through different tubing materials (28 cm). Percent cell recovery was determined by comparison of cell number recovered to expected cell number based on original cell concentration and volume. **(E–H)** DC retention in GOFlowChip prototypes with different well depths during cell loading, maintenance and recovery was assessed by cell count of the following outflow fractions: **(E)** MoDC loading at 1 ml/h, **(F)** 20-h culture with maintenance flow at 50 µl/h, **(G)** recovery wash after culture at 1.5 ml/min, and **(H)** MoDCs that remained in the GOFlowChip well after the recovery wash. Individual data points from experimental replicates (*n* = 3–5) and mean ± SEM are shown. *p ≤ 0.05, **p ≤ 0.01, determined by one-way repeated measures ANOVA with Geisser Greenhouse correction and Dunnett’s multiple comparisons test.

In our previous study, we designed the millifluidic GOFlowChip device (Figures 1A,B) to achieve long term external and luminal flow with single pluripotent stem cell-derived human intestinal organoids (Sidar et al., 2019). This device was adapted in the current study to optimize imaging of immune cell interactions with tissue-derived human gastric organoids. The GOFlowChip is composed of two layers, which were cut from clear cast polymethyl methacrylate (PMMA) 12″ x 12″ sheets (McMaster Carr, Elmhurst, IL, United States) using an automated laser cutter (Boss laser, Sanford, FL). A silicone rubber gasket was cut from 12″ x 12″, 1/16″ thickness silicone rubber sheets (Durometer 40A, White, McMaster Carr). For glass bottom and deep well GOFlowChips, the top and bottom layers were cut from PMMA sheets with thicknesses of 2.0 and 4.5 mm, respectively. To fabricate shallow well devices, the top layer was cut from 4.5 mm thick PMMA sheets, and the bottom layer was cut from 1.5 mm thick PMMA sheets. Blunt-tip stainless steel dispensing needles (90° angle, 20 gauge, McMaster Carr) were inserted into holes in the upper layer of the device for flow channel inlet and outlet and secured in place by 5 min epoxy application (Gorilla Clear Epoxy Adhesive, Gorilla Glue, Sharonville, OH, United States) from the outer facing section of the top layer. A glass coverslip (#1, 20 × 20 mm) was adhered to the bottom layer opening with epoxy (Gorilla Glue) to create a thin glass bottom window for imaging. The side ports that were part of the original GOFlowChip design were eliminated. Layers and gaskets were designed using AutoCAD software (Autodesk, San Rafael, CA, United States) and design files will be made available upon request. Device layers were sealed using the silicone gasket, and the assembled layers were compressed using nuts and bolts (McMaster Carr; 316 stainless steel, M3 x 0.3 mm thread, 10 mm length). For co-culture experiments, liquid flow was generated by a syringe pump (New Era Pump Systems, Inc., Farmingdale, NY, United States) and was directed across the channel and the central well where the organoids were placed.

### Extracellular Matrix Materials

We used Matrigel (#354234, Corning, Corning, NY), rat tail collagen type I (#354–236, Corning, Bedford, MA, United States) and commercially available synthetic hydrogels, VitroGel^®^ ORGANOID 1–4 (#VHM04-K, TheWell Bioscience, NJ) as extracellular matrix materials. VitroGels^®^ are xeno-free polysaccharide-based hydrogels designed for 3-D tissue cultures that have been used in a number of cancer studies (Pang et al., 2019; Haruna and Huang, 2020; Gebeyehu et al., 2021; Wang et al., 2021). The VitroGel^®^ ORGANOID (V-ORG) Discovery Kit includes four types of organoid hydrogels (V-ORG-1–4) which contain proprietary formulations of various bio-functional ligands and which have different mechanical strengths and degradability to fulfill the needs of different organoid culture conditions. The elastic moduli (G′) of the V-ORGs are around 50–300 Pa, similar to the elastic modulus of native ECM and of Matrigel (Haruna and Huang, 2020), with some differences between the four formulations. The elastic modulus was highest for V-ORG-3, followed by V-ORG-4, V-ORG-2 and V-ORG-1. After the initial screening, we selected V-ORG-3 (#VHM04-3, TheWell Bioscience, NJ) for further experiments.

### Organoid and Dendritic Cell Culture

Tissues for gastric organoid derivation were obtained from the National Disease Research Interchange or from Bozeman Health Deaconess Hospital with informed consent, using study protocols approved by the Institutional Review Board (IRB) of Montana State University. Gastric organoids were generated from isolated gastric glands using our previously published protocols (Sebrell et al., 2018; Sebrell et al., 2019). Morphology, growth characteristics and gene expression profiles of our gastric organoid model were described in our previous publication (Sebrell et al., 2018). Lentiviral transduction was used to generate mCherry-expressing organoids (Sebrell et al., 2019). For maintenance, organoids were cultured in Matrigel (#354234, Corning, Corning, NY) with L-WRN media: Advanced DMEM/F12 (#12634010, Gibco, Gaithersburg, MD, United States) with 10% FBS (#FB-02, Omega Scientific Tarzana, CA, United States), 2 mmol/L L-glutamine (#SH30034.1, Hyclone, Logan, UT), 100 U/L penicillin, 100 mg/L streptomycin (#15140122, Gibco, Waltham, MA, United States), 50 mg/ml gentamycin (#IB 2030; IBI Scientific, Peosta, IA), 0.25 ug/ml amphotericin B (#FG-70, Omega Scientific, Tarzana, California), 10 μM ROCK-inhibitor Y-27632 (#1254, Tocris Biosciences, Bristol, United Kingdom) and 10 μM TGF-β-inhibitor SB431542 (#1614, Tocris Biosciences), with 50% conditioned media from L-cells constitutively expressing Wnt3a, R-spondin 3, and noggin (Miyoshi et al., 2012). When organoids were cultured in V-ORG-1-4, each hydrogel was diluted 2:1 with 100% L-WRN-conditioned media to achieve a sufficient concentration of growth factors within the ECM.

To generate monocyte-derived DCs (MoDCs), blood samples were obtained with informed consent from healthy donors, using a study protocol approved by the Institutional Review Board (IRB) of Montana State University. Peripheral blood mononuclear cells (PBMCs) were isolated by density gradient centrifugation with Histopaque®-1077 (#10071, Sigma, Burlington, MA), followed by purification of CD14^+^ monocytes using anti-human CD14 MACS beads (#130–050–201; Miltenyi Biotec, Cologne, Germany), as previously described (Bimczok et al., 2011). Isolated monocytes were cultured in RPMI-1640 (#SH30027.01, Cytiva, Logan, UT) with 10% human AB serum (#35060CI; Corning, Manassas, VA), 100 U/L penicillin, 100 mg/L streptomycin (Gibco), 50 mg/ml gentamycin (IBI Scientific), 0.25 ug/ml amphotericin B (Omega Scientific), and 2 mmol/L L-glutamine (Hyclone) referred to here as “MoDC Media”. Monocytes were differentiated to MoDCs by culture with 7 ng/ml recombinant human (rh) IL-4 (#204-IL-050) and 25 ng/ml rh granulocyte-macrophage colony-stimulating factor (GM-CSF) (#215-GM-050, both R&D Systems, Minneapolis, MN). After 3–4 days in culture, MoDCs were recovered by vigorous pipetting followed by washing with PBS.

### GOFlowChip Cell Delivery and Recovery Experiments

The following protocol was used for loading of MoDCs into the GOFlowChip: First, the device was soaked in ethanol for 5 min, air-dried in a biosafety hood for 10 min and then assembled. 10–12 cm of tubing was attached at the inlet and outlet, then tubing and device were washed by syringe pump (New Era Pump Systems, Inc., Farmingdale, NY, United States) delivery with 3 ml PBS at a flow rate of 1 ml/min using 3 ml Luer lock syringes (#309657, BD Biosciences, Franklin Lakes, NJ, United States). Next, the device was coated with 3 ml of fetal bovine serum (FBS, Omega Scientific) at a flow rate of 600 µl/min to reduce nonspecific binding of MoDCs to the tubing or device, washed again with PBS to remove excess FBS, then equilibrated with 3 ml MoDC media at a flow rate of 1 ml/min. MoDCs were loaded into the GOFlowChip at a flow rate of 1 ml/h for 30 min, at a concentration of 10^6^ cells/ml. The syringe pump was elevated vertically during cell loading to prevent settling of cells within the syringe. For culture in the chip, the maintenance flow of 50 µl/h was used, as it has been calculated in our previous work to overturn the media volume every 2 days, replicating conventional culture protocols used with HGO cultures maintained on standard well plates. MoDCs were removed from the GOFlowChip by flushing the device (recovery wash) at a rapid flow rate of 1.5 ml/min for 5 min with a 10 ml Luer lock syringe (#20180914, Fisher Scientific, Pittsburg, PA, United States) using PBS supplemented with 1% FBS and 2 mM EDTA (#E177, Amresco, Solon, OH, United States).

To optimize tubing materials for cell delivery, 28 cm of each tubing type, not connected to the device, was coated and equilibrated, then MoDCs were pumped through each tubing in parallel, following the GOFlowChip loading protocol. The following tubing materials were assessed: polyethylene (#BB31695-PE15), silicone (#BB519-13), PTFE (#BB311-22), and polyurethane (#BB520-65), all from Scientific Commodities Inc., Lake Havasu City, AZ, United States. After the loading sequence, cell recovery from the tubing outflow was determined by hemocytometer cell count and comparison to the expected cell yield, based on the cell concentration that was loaded. To select the optimal GOFlowChip well depth, MoDCs were loaded into either a 1.5 mm shallow well or a 4.5 mm deep well GOFlowChip using the fluidics protocol described above. To compare loading efficiency, the loading outflow for each well was collected over 30 min. The MoDCs were then cultured overnight on the device, and the maintenance fraction was collected over 16–18 h. MoDCs were then flushed from the well, and the outflow was collected over 5 min. The device was disassembled, and the MoDCs remaining in the well (well fraction) were removed by washing with PBS. Cell yield of the loading, maintenance, recovery wash and well fractions was determined by hemocytometer count and comparison to the cell numbers originally loaded onto the device.

### Transwell^®^ Migration Assays

For cell migration experiments, MoDCs were layered on 96-well corning 8 µm pore Transwells^®^ (#3374, Corning, Kennebunk, ME, United States) coated with 20 µl of gelled ECM material, or with no ECM material for comparison. Experiments were performed in duplicate. Transwells were equilibrated with MoDC media for 1 h at 37°C, 5% CO_2_, then MoDCs were plated at the apical side of the Transwell^®^ at 2 million cells/ml in MoDC media and incubated at 37°C, 5% CO_2_ for 3 h. The manufacturer’s recommended volumes of 75 µl for the apical side and 235 µl for the basolateral chamber were used for all experiments. To induce chemotaxis, the basolateral media was supplemented with 8 ng/ml of CXCL1 (#300–11, PeproTech, Cranbury, NJ, United States), a known human MoDC chemoattractant (Sebrell et al., 2019), or bovine serum albumin (BSA) (CAS#9048–46–8, Fisher Bioreagents, Fairlawn, NJ, United States) for validation of chemotaxis through Matrigel (Corning). After the 3-h incubation, CellTiterGlo^®^ reagent (#G9242, Promega, Madison, WI, United States), which produces luminescence in response to live cells, was used to quantify the cells that had migrated to the basal compartment of the Transwell^®^. Plates were analyzed on a luminescence plate reader and data are shown as relative light units (RLUs). Transwell coatings were prepared as follows: for collagen-Matrigel mixtures, 3.8 mg/ml rat tail type I collagen was combined with Matrigel (Corning) at ratio of 1:3, 1:1, and 3:1, then gelled for 15 min at 37°C, 5% CO_2_. Whole Matrigel or Matrigel diluted using MoDC media and was gelled for 15 min at 37°C, 5% CO_2_. V-ORGs 1-4 were diluted 1:2 with MoDC media, per manufacturer instructions, then allowed to gel at room temperature for 15 min prior to addition of the DCs. For migration inhibition experiments, the chemotaxis assay was repeated as above, but MoDCs were resuspended in MoDC media containing 2 μM Actinonin (#A6671-10 MG, Millipore Sigma, Burlington, MA, United States), a broad spectrum MMP inhibitor, or DMSO as a solvent control immediately before addition to the Matrigel-coated Transwell^®^.

### Cell Viability and MoDC Maturation Experiments

Viability of organoids and MoDCs was determined by fluorescence activated cell sorting (FACS) after staining with the Fixable LIVE/DEAD yellow stain kit, 405 nm excitation (#L34967, Thermo Fischer Scientific, Wilhelm, MD, United States) or a panel of antibodies for MoDC maturation markers as follows at a dilution of 1:50: HLA-DR eF450 (#48–9952-42CD86, eBioscience, San Diego, CA, United States), CD86 APC (#555660, BD Biosciences, San Jose, CA, United States), CD83 PE (#556855, BD Biosciences) and CD80 FITC (#555683, BD Biosciences). For organoid viability experiments, HGOs were passaged normally with trypsin, then added into the V-ORG 1-4 matrices following the manufacturer protocol or into Matrigel and cultured for 5 days. To measure HGO growth in each ECM material, the diameters of ten organoids fully formed 1 day after plating (Day 1) were imaged and measured on days 1, 3, and 5 of culture. At day 5, the media was removed, and each culture was removed from the well by washing with cold PBS. Trypsinization was used to break down organoids into single cells for live/dead staining and analysis.

For MoDC V-ORG viability and maturation experiments, MoDCs were layered above gelled V-ORG-1-4, Matrigel or cultured alone with no ECM for 20 h at a concentration of 10^6^/ml. For viability experiments a 96-well plate format was used, and for maturation experiments, a 48-well plate was used, to improve total endpoint cell count for accurate assessment of DC maturation status. To collect MoDCs for cell staining, the unattached cells in the media were collected first, then MoDCs attached or embedded within each matrix were collected by dilution with ice cold PBS and vigorous pipetting and scraping. Cells and remaining ECM were pelleted by centrifugation and then diluted once more with 1 ml of ice-cold PBS before staining.

To determine viability and maturation state of MoDCs cultured within the GOFlowChip, MoDCs were loaded into the device using the previously described loading protocol, cultured with maintenance flow for 0.5–3 h or overnight (16 h), then recovered from the device for collection. In parallel, control MoDCs were cultured in 24-well tissue culture plates. MoDCs were stained for dead cells as above, then stained with the above antibody panels for the DC maturation. FACS data was analyzed using FlowJo™ software (TreeStar, Ashland, OR, United States).

### Organoid-Dendritic Cell Co-Cultures on the GOFlowChip

The following final protocol was utilized to prepare GOFlowChip co-cultures of MoDCs and HGOs. A complete list of materials and reagents is provided in Table 1.

1) Preparation of HGOs for GOFlowChip culture:
  a Culture organoids in Matrigel on 24-well-plates for 4–6 days using standard protocols (Miyoshi and Stappenbeck, 2013; Sebrell et al., 2018).
  b Remove media from one or two wells of HGOs.
  c Add 750 μl of Cell Recovery solution per well (#354253, Corning, Glendale, AZ, United States).
  d Gently scrape Matrigel patty with organoids from the well using a wide bore tip. Transfer to a microcentrifuge tube.
  e Incubate on ice in a total volume of Cell Recovery Solution of 1.5 ml for 30 min. After incubation, the organoids will pool to the bottom of the tubes. Remove Cell Recovery Solution.
  f Wash organoids twice with ice cold PBS. Pellet by gentle centrifugation at 100 *g* for 2 min.
2) Preparation of MoDCs:
  a Prepare culture of MoDCs from blood monocytes using standard protocols (Sebrell et al., 2019; Roe et al., 2020).
  b Harvest MoDCs by vigorous pipetting and washing with PBS.
  c Stain MoDCs with CellTracker™ Green CMFDA dye (#C7025, Invitrogen, Waltham MA, United States) following the manufacturer’s protocol.
  d Set cell concentration to 10^6^/ml in 2–3 ml MoDC medium.
  e Filter DCs through a 70 μm cell strainer to remove cell clumps.
3) Preparation and assembly of the GOFlowChip:
  a Disinfect the glass-bottom GOFlowChip by soaking in PBS with penicillin and streptomycin for 5 min
  b Dry chip thoroughly within a biosafety hood for 15 min.
  NOTE: Ethanol cannot be used to sterilize the glass bottom GOFlowChip, as it disturbs the epoxy used to adhere the glass cover slip, which can cause leakage over time.
  c After ensuring that no PBS remains in the well, pipette 30–40 µl of V-ORG-3 into the well
  NOTE: The well must be completely dry before addition of V-ORG-3, or the gel will not adhere to the glass.
  d Immediately add the prepared organoids and allow matrix to gel.
  e Assemble GOFlowChip by attaching the top PMMA layer with screws.
  f Attach tubing. For attachment of inlet tubing to the device, attach a syringe of PBS to the tubing and fully fill the tubing before attachment, to prevent any bubble formation.
4) Loading of DCs onto the GOFlowChip:
  a Perform loading sequence described to add DCs.
  b Maintain GOFlowChip at 37°C with media flow at 50 µl/h for up to 72 h.

**TABLE 1:** Formulations, materials, and equipment.

L-WRN medium (for organoid growth)
-Advanced DMEM/F12 (#12634010, Gibco, Gaithersburg, MD, United States)
-50% conditioned media from L-cells constitutively expressing Wnt3a, R-spondin 3, and Noggin
-10% FBS (FB-02, Omega Scientific Tarzana, CA, United States)
-2 mmol/L L-glutamine (#SH30034.1, Hyclone, Logan, UT)
-50 mg/ml gentamycin (#IB 2030; IBI Scientific, Peosta, IA)
- 100 U/L penicillin, 100 mg/L streptomycin (#15140122, Gibco, Waltham, MA, United States)
-0.25 ug/ml amphotericin B (# FG-70, Omega Scientific, Tarzana, CA)
-10 μM ROCK-inhibitor Y-27632 (#1254, Tocris Biosciences, Bristol, United Kingdom)
-TGF-β-inhibitor SB431542 (1614, Tocris Biosciences)
**MoDC medium (for MoDC culture and MoDC-HGO Co-culture)**
-RPMI-1640 (#SH30027.01, Cytiva, Logan, UT)
-10% human AB serum (#35060CI; Corning, Manassas, VA)
-100 U/L penicillin, 100 mg/L streptomycin (Gibco), 50 mg/ml gentamycin (IBI Scientific)
-0.25 ug/mL amphotericin B (Omega Scientific)
-2 mmol/L L-glutamine (Hyclone)
-7 ng/ml recombinant human (rh) IL-4 (#204-IL-050, R&D systems, Minneapolis, MN) *for MoDC differentiation only*
-25 ng/ml rh granulocyte-macrophage colony-stimulating factor (GM-CSF, #215-GM-050, R&D systems, Minneapolis, MN). *For MoDC differentiation only*
**Materials and equipment**
-24-Well tissue culture plates
-3 ml Luer lock syringes (#309657, BD Biosciences, Franklin Lakes, NJ, United States)
-Histopaque®-1077 (#10071, Sigma, Burlington, MA)
-Anti-Human CD14 MACS beads (#130–050–201; Miltenyi Biotec, Cologne, Germany)
-Wide bore pipette tips
-Cell recovery solution (#354253; Corning, Manassas, VA)
-Celltracker™ Green CMFDA dye (#C7025, Invitrogen, Waltham, MA)
-70 μm cell strainer
-VitroGel^®^ ORGANOID-3 (#VHM04-3, TheWell Bioscience, NJ)
-Silicone tubing (#BB519-13, Scientific Commodities Inc.)
-Syringe pump (New Era Pump Systems, Inc., Farmingdale, NY)

### Confocal Imaging and Image Analysis

Confocal time-lapse imaging at 10x magnification was performed on a Leica SP5 Confocal Scanning Laser Microscope (Leica, Wetzlar, Germany) with 405, 488, 561 and 633 nm laser excitation lines and a heated stage with an environmental control chamber. Movements of the MoDCs and their interactions with the HGOs were tracked over 20 h. The Z-step was 11 µm and images were taken every 10 min. Co-cultures with V-ORG-3 and Matrigel were each repeated twice. For quantitative assessment of MoDC associations with HGOs for each ECM (Kelsall and Rescigno, 2004), average distance of MoDCs to an organoid and (Mann and Li, 2014) number of MoDCs within 75 µm were determined for each experiment. Five randomly selected Z sections containing at least one HGO were selected for assessment of average distance, and ten randomly selected Z sections were assessed for number of MoDCs 75 µm from an HGO. To avoid analyzing MoDCs in a co-culture area more than once, Z slices selected were all at least three Z-sections apart. One Matrigel experiment could not be analyzed for average distance of MoDCs to HGOs, as no MoDCs penetrated the Matrigel far enough to reach any HGOs. The particle tracking software Imaris (Bitplane, Concord, MA) was used for generation of time lapse videos and images of MoDC movement. To quantify penetration of MoDCs into each ECM material after introduction at the surface, the instantaneous velocity of each detected MoDC moving in the negative Z direction for each 10 min time point was determined using Imaris. The number of MoDC -Z tracks analyzed for Matrigel and V-ORG-3 were 6,746 and 21,089 events, respectively.

### Statistical Analyses

All data were analyzed using GraphPad version 9.1.2 (San Diego, CA). Data are shown as mean ± SEM. The Student’s *t* test or a one- or two-way ANOVA with Tukey’s or Dunnett’s multiple comparisons test was used to determine statistical significance. Differences were considered significant at *p* ≤ 0.05.

## Results

### Optimization of the Millifluidic GOFlowChip Platform for Dendritic Cell Loading and Maintenance

The initial step for integration of monocyte-derived dendritic cells (MoDCs) into the GOFlowChip (Figures 1A,B) was to determine the optimum conditions for loading of MoDCs into the device. During MoDC loading experiments, we detected potential adhesion of MoDCs to polyethylene tubing (PE), a common tubing material in fluidics studies that we used in our previous experiments (Miron-Mendoza et al., 2010; Chen et al., 2013; Chang et al., 2017). Because high adhesion of the DCs to tubing would decrease cell delivery into the GOFlowChip, we investigated the adherent properties of MoDCs to multiple tubing materials; PE, silicone, polytetrafluoroethylene (PTFE), and polyurethane (PU). MoDCs were flowed through different tubing materials at 1 ml/h, and cell recovery was compared. The tubing length was increased from 10 to 28 cm to exaggerate differences between each material. Cell yield was lowest for PE and PU tubing, with only 10.0 ± 2.7% and 20.0 ± 4.5% of MoDCs recovered. Both silicone and PTFE tubing resulted in significantly higher MoDC recovery then the PE, with 37.0 ± 7.1% (*p* ≤ 0.05) and 51.5 ± 7.6% (*p* ≤ 0.01), respectively (Figure 1C). To investigate whether adhesion to these materials would be similar for other immune cells, we also analyzed blood lymphocytes, referred to here as CD14^–^PBMCs. Cell loading of CD14^–^ PBMCs was less affected by tubing material, though increased yields compared to PE were also found when using PTFE (*p* ≤ 0.05) and silicone (Figure 1D). While PTFE resulted in the lowest cell retention, its rigidity made it impractical for GOFlowChip experiments and imaging. Therefore, silicone tubing was selected for further experiments.

We next evaluated the impact of well depth on MoDC loading and retention within the modified GOFlowChip, using devices with either a 1.5 mm shallow well or a 4.5 mm deep well. MoDCs were loaded into each device for 30 min at a cell concentration of 1 million/ml, at 1 ml/h using a syringe pump, and then were cultured overnight with a previously determined maintenance flow rate of 50 µl/h. The loading and maintenance outflow were collected to recover any MoDCs extruded from the device (Figures 1E,F). After culture, MoDCs were recovered from the wells using a rapid flow rate of 1.5 ml/min (recovery wash, Figure 1G), then the device was disassembled and any remaining MoDCs within the well were collected (Figure 1H). While not statistically significant, compared to the 1.5 mm device, the 4.5 mm deep well GOFlowChip reduced the number of MoDCs lost during loading and maintenance flow (Figures 1E,F), and increased the recovery of MoDCs during the recovery wash and the recovery of cells in the well at the experimental endpoint (Figures 1G,H). Therefore, the remaining experiments were performed with the deep well GOFlowChip.

### The GOFlowChip Does not Affect Dendritic Cell Viability or Maturation

**FIGURE 2:**
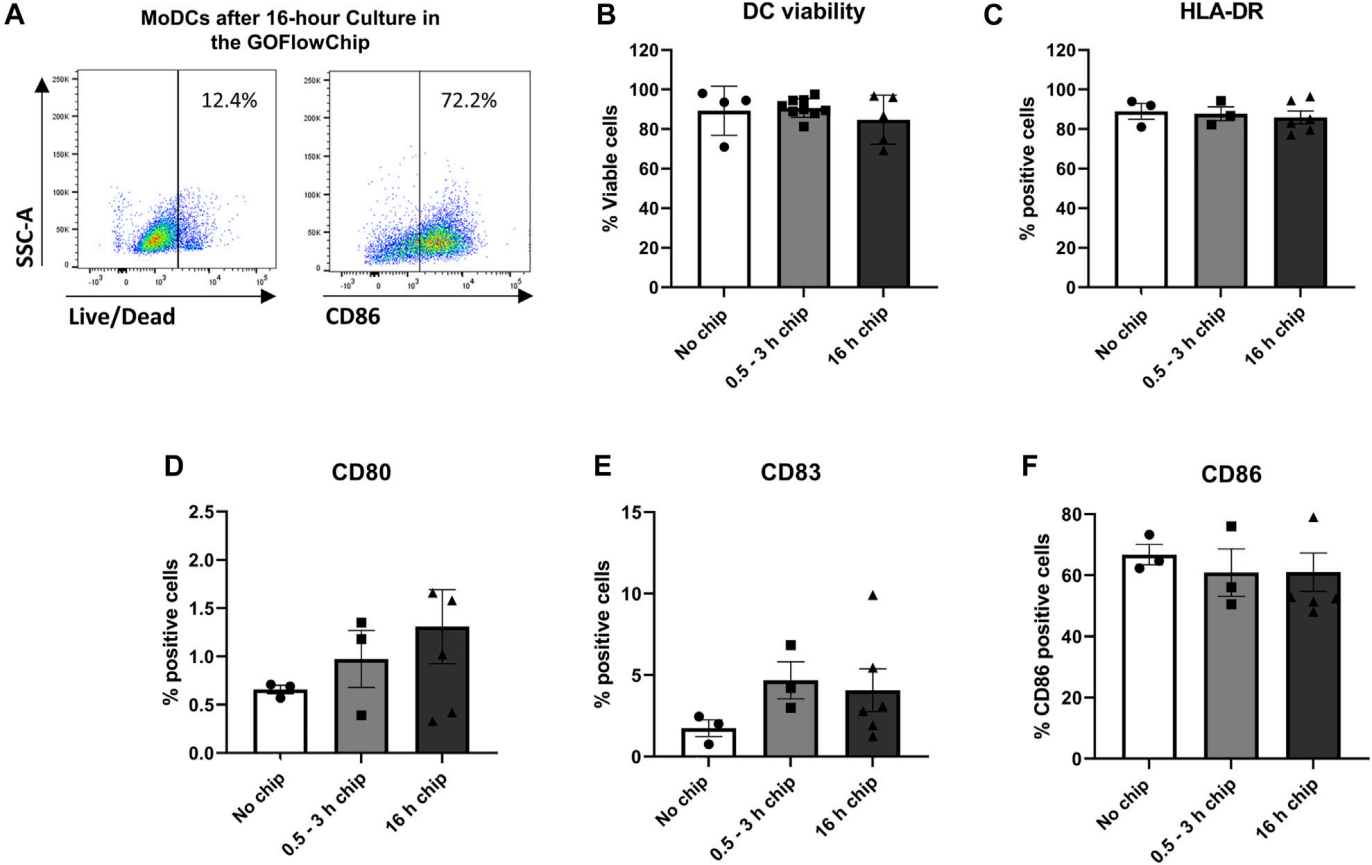
Prolonged culture on the GOFlowChip does not significantly affect MoDC viability and maturation. MoDCs were cultured within the GOFlowChip for 0.5–16 h and then were assessed for viability and expression of DC activation markers by flow cytometry. **(A)** Representative density plots of MoDC viability (left panel) and maturation (CD86, right panel) after overnight culture within the GOFlowChip. Pooled data from multiple experiments showing **(B)** viability (LIVE/DEAD yellow stain) and expression of **(C)** HLA-DR, **(D)** CD80, **(E)** CD83, and **(F)** CD86. *N* = 3-6, one-way ANOVA, individual values, mean ± SEM are shown.

A previous report showed that certain organic polymers used in tissue chips such as PDMS or PTFE can impact DC function (Roch et al., 2016). Therefore, we sought to confirm that culture on the GOFlowChip device had no negative impact on DC viability and maturation. MoDCs were loaded into the GOFlowChip at a concentration of 1 million cells/ml for 30 min. After 0.5–3 h or overnight culture (16 h), MoDCs were removed from the GOFlowChip using cold PBS buffer supplemented with 1% FBS and 2 mM EDTA using a rapid flow rate of 1.5 ml/min for 5 min. MoDC viability was determined via flow cytometry (Figure 2A). After 3 h or overnight culture within the GOFlowChip, viability was not significantly reduced when compared to MoDCs cultured outside the device, with ∼90% live cells seen with all three experimental conditions (Figure 2B). In parallel experiments, we assessed the activation state of the DCs following culture on the GOFlowChip. As shown in Figures 2A,C–F, expression of DC maturation markers HLA-DR, CD86, CD83 and CD80 did not increase after culture within the GOFlowChip.

### The Synthetic Hydrogel VitroGel^®^ ORGANOID Allows Improved Chemotactic Motility of MoDCs Compared to Matrigel

To analyze DC interactions with co-cultured organoids, we previously relied on spontaneous DC recruitment through the Matrigel in response to organoid-derived chemokines such as CXCL1 (Sebrell et al., 2019). However, this process was not very efficient. Most DC-organoid interactions were limited to organoids closer to the Matrigel surface, while organoids embedded deeply within the Matrigel often failed to recruit DCs (data not shown). To facilitate interactions of MNPs with the epithelium of our co-culture system, we next analyzed whether modifying the ECM would lead to increased DC migration toward the organoids while still supporting organoid growth and organoid and DC viability.

**FIGURE 3:**
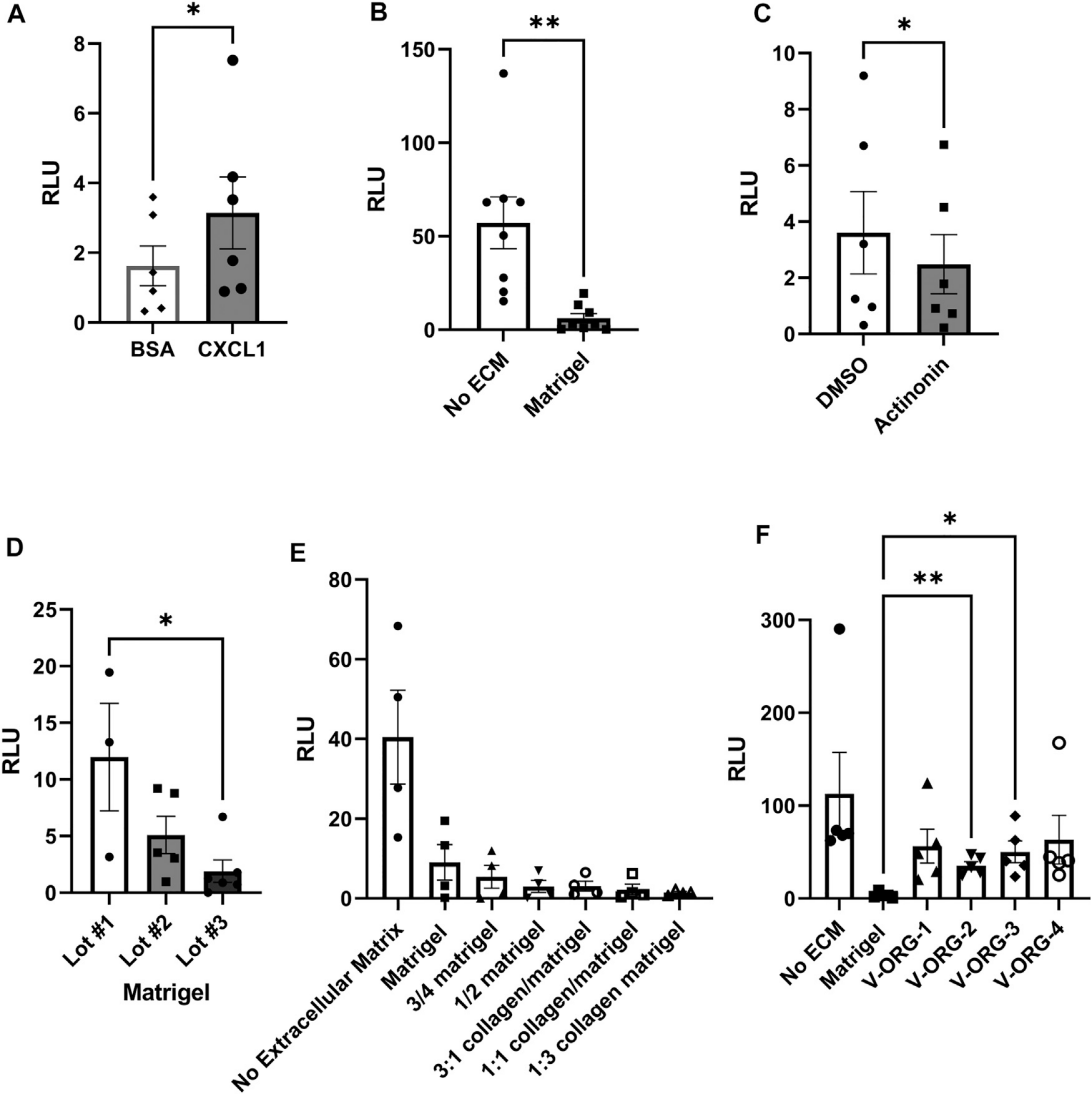
MoDC chemotactic migration is improved by VitroGel^®^ ORGANOID synthetic hydrogels. For analysis of MoDC chemotactic migration through extracellular matrix (ECM) materials, 8 µm pore Transwell^®^ inserts were coated with each ECM, then MoDCs were plated at the apical side, with the chemoattractant CXCL1 (8 ng/ml) added to the basolateral compartment. DC migration to the basolateral compartment of the Transwell^®^ was determined by using the CellTiterGlo^®^ reagent, measured as relative light units (RLU). **(A)** Confirmation of MoDC chemotaxis through a Matrigel layer by the chemokine CXCL1, with bovine serum albumin (BSA) used as a protein control (*n* = 6). **(B)** Comparison of CXCL-1-induced MoDC migration through uncoated and Matrigel-coated Transwell^®^ membranes (*n* = 8 independent experiments). **(C)** Treatment of MoDCs with the broad-spectrum matrix metalloproteinase inhibitor actinonin (2 µM) reduced chemotaxis through Matrigel. **(D)** Migration of MoDCs through different lots of Matrigel demonstrates significant batch-to-batch variation. **(E)** Assessment of MoDC chemotaxis through diluted Matrigel and Matrigel-collagen mixtures (*n* = 3). **(F)** MoDC chemotaxis through VitroGel^®^ ORGANOID 1–4 (V-ORG; *n* = 5) compared to Matrigel. Data were analyzed by two-tailed Wilcoxon test **(A–C)** or one-way ANOVA **(D–F)**, *p ≤ 0.05, **p ≤ 0.01. Individual datapoints, mean ± SEM are shown.

We used a Transwell^®^ chemotaxis assay to compare different matrix materials for their ability to allow MoDC migration. The apical membranes of the Transwell^®^ inserts were coated with ECM materials and MoDCs were placed on top. CXCL1, a DC chemoattractant expressed in the gastric epithelium that was previously reported by our group to attract MoDCs (Sebrell et al., 2019), was added to the basolateral chamber. Chemotaxis was evaluated after 3 h using the CellTiterGlo^®^ reagent. MoDCs showed significant chemotaxis towards CXCL1 (8 ng/ml) across the Matrigel-coated Transwell^®^ inserts (Figure 3A, *p <* 0.05). However, migration through Matrigel was significantly decreased when compared to transmigration through uncoated Transwells (Figure 3B, *p <* 0.01). We also established matrix metalloproteinase activity as a potential mechanism for chemotaxis through Matrigel, because MoDC migration was further inhibited with the cells were treated with the broad spectrum MMP inhibitor actinonin (Figure 3C). Of note, migration of MoDCs through Matrigel was significantly affected by batch-to-batch variation (Figure 3D). To evaluate alternative ECM materials, we first assessed Matrigel-collagen mixtures and diluted Matrigel to determine if a less dense matrix would increase MoDC migration. While collagen has been reported to enhance motility of cancer cells (Anguiano et al., 2017), the addition of collagen dramatically decreased the chemotactic ability of MoDCs through the matrix, with almost no cells detected in the basolateral chamber (Figure 3E). Interestingly, dilution of Matrigel with tissue culture media also reduced MoDC chemotactic ability (Figure 3E). We then assessed synthetic hydrogels as possible ECM replacements. We obtained four xeno-free polysaccharide-based hydrogels, specifically designed for culture of 3D organoids, VitroGel^®^ ORGANOID-1, 2, 3, and 4 (subsequently abbreviated as V-ORG), which differ in the presence of functional ligands and degradability. All four hydrogels greatly improved chemotactic migration of MoDCs compared to Matrigel, with V-ORG-2 and V-ORG-3 achieving statistical significance (Figure 3F), making them promising candidates for HGO-MoDC co-cultures within the GOFlowChip.

### VitroGel^®^ ORGANOID Hydrogels Successfully Maintain Viable Human Gastric Organoid and Dendritic Cell Cultures

**FIGURE 4:**
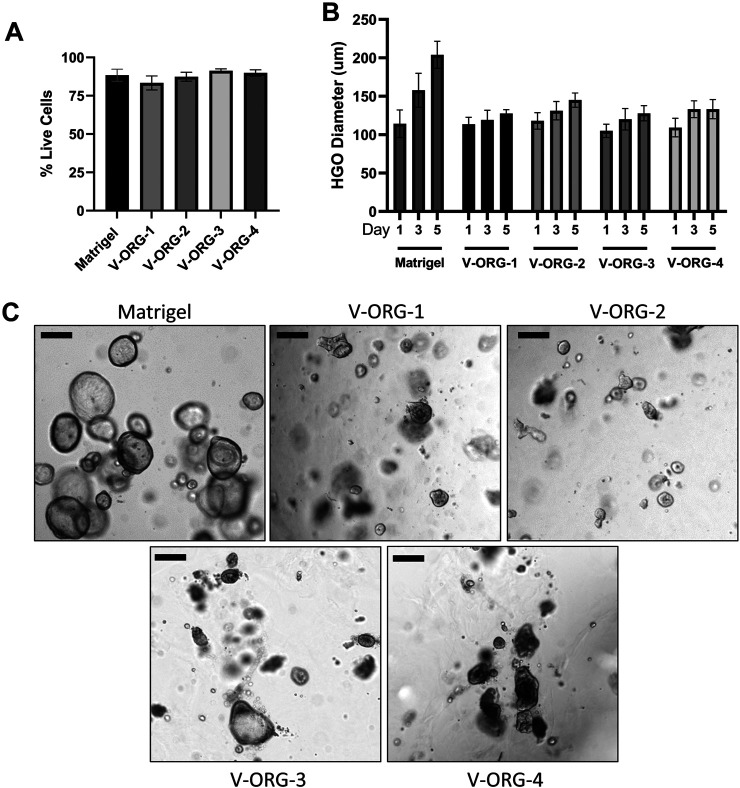
Human gastric organoids remain viable when cultured in VitroGel^®^ ORGANOID. HGOs were cultured in V-ORG-1-4 or Matrigel for up to 5 days and then analyzed for viability and growth by flow cytometry and microscopy. **(A)** HGOs were stained with LIVE/DEAD Yellow and analyzed for cell death by flow cytometry after 5 days. **(B)** HGOs were passaged into V-ORG-1-4 or Matrigel. At days 1, 3, and 5 of culture, the maximum diameter of ten fully formed organoids was measured to compare growth in the different ECM materials. Bars show mean ± SEM of three independent experiments. Two-way ANOVA determined a significant effect of time (*p =* 0.0005) and ECM material (*p =* 0.0008) on HGO diameter. **(C)** Representative images of HGOs maintained in the different ECM materials after 4 days. Bar = 200 µm.

Having determined that the V-ORG hydrogels improved MoDC migration, the viability and growth of HGOs cultured within the hydrogels was analyzed. To that end, HGOs were cultured within V-ORG-1-4 or Matrigel for 5 days. All V-ORGs supported HGO culture, with no significant decrease in cell viability in a FACS-based viability assay (Figure 4A). However, some morphological changes were observed. To compare growth of HGOs in V-ORGs, the diameter of ten organoids from each growth condition were measured on days 1, 3, and 5 of culture. The organoids continued to grow in the V-ORG, but growth appeared less robust compared to HGO growth in Matrigel. HGOs in the synthetic hydrogels generally reached 100–200 µm in diameter after 5 days, while HGOs cultured in Matrigel continued growth, sometimes reaching >300 µm in diameter **(** Figures 4B,C). Two-way ANOVA revealed that both time and ECM material significantly influenced organoid size (*p* ≤ 0.001). Interestingly, HGOs grown in V-ORG 1-4 also had less defined spherical shapes and darker centers after 4 days (Figure 4C). Similar morphological changes have previously been associated with increased organoid differentiation (Schlaermann et al., 2016). Since larger organoids (>400–500 µm) are required for microinjections to study bacterial infection and since we cannot exclude that prolonged culture of human gastric organoids in V-ORG hydrogels causes functional changes, we established a protocol where we cultured the organoids in Matrigel until they had reached the desired size and then transferred the organoids to V-ORG for a 20 h co-culture experiment. Maintenance of organoids in V-ORG-3 for only 20 h did not result in overt morphological changes (Supplementary Figure S1).

**FIGURE 5:**
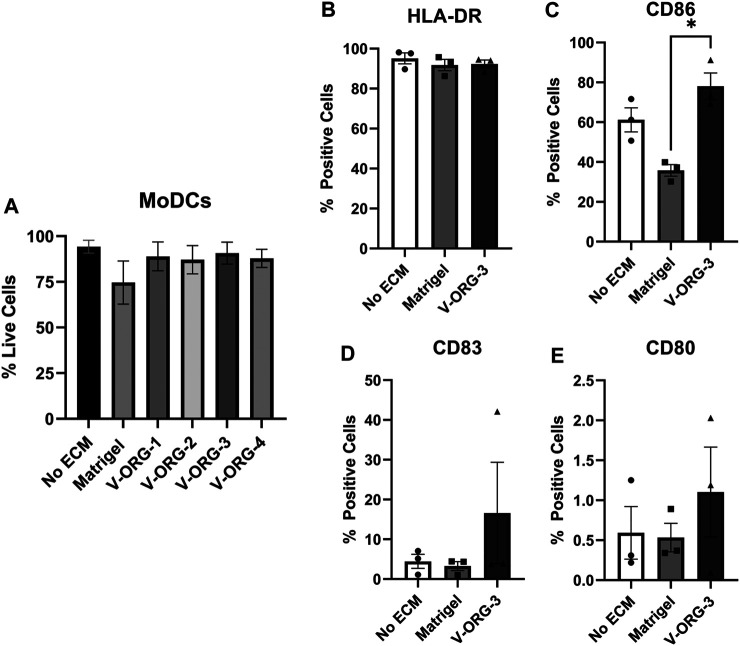
MoDCs cultured with VitroGel^®^ ORGANOID maintain viability and show minimal differences in DC maturation. MoDCs were plated onto each ECM material (Matrigel or V-ORG) or directly into the well (No ECM), cultured for 20 h, then stained with **(A)** LIVE/DEAD Yellow or with antibodies to **(B)** HLA-DR, **(C)** CD86, **(D)** CD83 and **(E)** CD80 for analysis by flow cytometry; (*n* = 3 independent experiments). Bars depict mean ± SEM; Statistical analysis was performed by one-way ANOVA, *p ≤ 0.05.

We then analyzed whether V-ORGs affected MoDC viability or maturation status. MoDCs were layered above Matrigel- or V-ORG-coated wells, cultured for 20 h to mimic the GOFlowChip co-culture, and then were assessed for cell viability by FACS. MoDCs remained 80–90% viable when cultured with all of the four V-ORG hydrogels (Figure 5A), and interestingly, their viability was improved compared to culture with Matrigel, though the difference was not statistically significant. We selected V-ORG-3 for further experiments, because it enabled significantly improved DC migration as well as excellent DC and organoid viability. To determine the effect of ECM materials on DC phenotype, MoDCs were cultured with Matrigel, V-ORG-3, or without ECM and then were analyzed for expression of HLA-DR, CD86, CD83, and CD80, as in Figure 2. Interestingly, MoDC culture with Matrigel slightly decreased the expression of maturation markers compared with MoDC culture in the absence of ECM, whereas culture in V-ORG-3 caused a small increase in maturation marker expression (Figures 5B–E). However, these trends were generally not significant.

### VitroGel^®^ ORGANOID 3 Enhances MoDC Migration and Association With HGOs on the GOFlowChip

**FIGURE 6:**
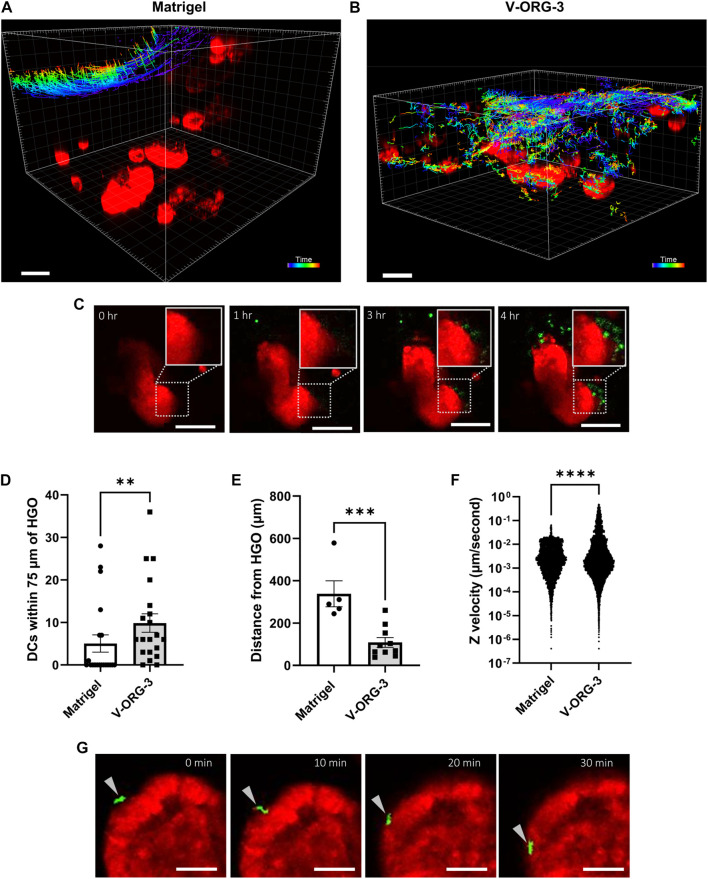
VitroGel^®^ ORGANOID 3 enables improved MoDC migration and MoDC-HGO interactions on the GOFlowChip. For GOFlowChip experiments, HGOs were recovered from standard Matrigel cultures on 24-well plates using Cell Recovery solution and then were layered onto either Matrigel or V-ORG-3 that had been placed into the central well of the GOFlowChip. MoDCs were introduced into the system by syringe pump. The co-culture was imaged using live confocal microscopy for 20 h. **(A)** Migration tracks of MoDCs (colored lines) moving through Matrigel or **(B)** V-ORG-3 and associating with HGOs (red). Bar = 200 μm; track colors represent different time points after MoDC introduction. **(C)** Representative time-lapse confocal images show that MoDCs (green) accumulate close to HGOs (red) embedded in V-ORG-3 within 3–4 h, bar = 100 µm. **(D,E)** To quantify DC associations with HGOs in each ECM, we determined **(D)** the number of MoDCs within a 75 µm radius around the HGOs and **(E)** the average distance between DCs and the nearest HGOs after 20 h using 5–10 randomly selected Z planes from two separate experiments. **(F)** Velocity of single MoDCs in the GOFlowChip in the vertical Z-direction, representing cell migration into the ECM, was determined by particle tracking; *n* ≥ 6,000 events; data were analyzed by Mann Whitney *U* Test ****p ≤ 0.0001. **(G)** An MoDC (arrowhead) attaches to and migrates along the basolateral surface of an HGO over 30 min, bar = 50 µm.

Lastly, after confirming that HGOs and DCs remained viable within V-ORG-3 and that MoDCs could easily migrate through V-ORG-3, we compared co-cultures of MoDCs and HGOs embedded within either V-ORG-3 or Matrigel using time lapse confocal microscopy. HGOs were transferred from 24-well plates into the central well of the GOFlowChip, and MoDCs were introduced through the inlet channel at 1 ml/h for 30 min, followed by 20 h of maintenance flow at 50 µl/h, as described earlier. As shown in Figure 6A and Supplementary Video S1, little migratory activity of MoDCs towards organoids embedded deep in the ECM was observed for Matrigel, with most DCs remaining on the Matrigel surface throughout the 20 h experiment. In contrast, most MoDCs were able to penetrate up to 500 µm into the V-ORG-3 matrix and accumulate in the proximity of the HGOs (Figure 6B; Supplementary Video S2), and large numbers of MoDCs were found directly adjacent to V-ORG-3-embedded HGOs after 3–4 h of culture (Figure 6C).

To quantify differences in chemotactic motility of MoDCs within the two ECM materials, randomly selected Z-planes at the endpoint of each experiment were compared for average distance of MoDCs to HGOs, and the number of MoDCs within a 75 µm radius of HGOs was quantified. Culture with V-ORG-3 led to a significant increase in the number of MoDCs found in close proximity to the HGOs (Figure 6D) and a significant decrease in average MoDC-HGO distance after 20 h of culture (Figure 6E). Conversely, in half of Matrigel endpoint Z-planes, no MoDCs associated with HGOs, confirming the motility constraints of Matrigel. Though two replicate experiments were performed per ECM, one Matrigel replicate could not be evaluated for average distance of MoDCs from HGOs as the MoDCs remained almost entirely on the Matrigel surface, never reaching the depth of any HGO. Cell tracking in the time lapse series also confirmed improved DC motility in the synthetic hydrogel matrix. Thus, MoDC velocity within the ECM in the negative Z-direction, indicating vertical cell movement from the ECM surface towards the bottom of the well, was significantly increased (*p* ≤ 0.0001) on GOFlowChip cultures with V-ORG-3 compared to Matrigel (Figure 6F). Importantly, individual DCs that were migrating along the basolateral surface of the HGOs could be observed in tissue chips containing V-ORG-3, consistent with physiological DC immunosurveillance activity (Figure 6G). These experiments demonstrate the feasibility of immune cell-organoid co-cultures on a tissue chip and show that the synthetic hydrogel matrix material V-ORG-3 supports spontaneous physiological DC recruitment, DC-epithelial interactions and immunosurveillance.

## Discussion

We previously developed a millifluidic chip platform, the GOFlowChip, for culture of 3-D organoids under long term flow conditions (Sidar et al., 2019). As tissue chip technology progresses, researchers are working to increase the complexity of organoid-based MPSs by incorporating immune system components (Morsink et al., 2020). Here, we adapted the GOFlowChip for integration of MoDCs to model MNP interactions with the gut epithelium in real-time.

One major challenge when culturing cell types from different tissues in one system is that optimum culture conditions for each cell type may differ significantly. In our previous study, we showed that co-cultures of human gastric organoids and MoDCs could be maintained for at least 48 h in culture media optimized for human MoDCs (Sebrell et al., 2019). However, to establish co-cultures on a tissue chip, the chip materials also needed to be tested for their compatibility with the DCs. Polydimethylsiloxane (PDMS), a commonly used biocompatible polymer for MPS devices has been reported to induce MoDC maturation and demonstrates some problematic absorption of hydrophobic small molecules (Toepke and Beebe, 2006; Roch et al., 2016). We verified that culture within our PMMA device did not induce activation and maturation of MoDCs, allowing the study of potentially inflammatory disease states or therapies without interference. Other functional properties of immune cells should also be investigated during integration into MPSs, such as cell adherence to device materials. We demonstrated up to a three-fold decrease in cell delivery of MoDCs and of blood lymphocytes based on differences in tubing material. Immune cell adhesion was lowest in PTFE tubing, but as PTFE is very rigid and has also been reported to affect DC function (Roch et al., 2016), silicone was selected for our MPS platform, since DC adhesion to this material also was relatively low.

Selection of a functional and physiological ECM also is necessary for the development of complex *in vitro* experimental systems, because the biochemical and mechanical properties of ECMs heavily influence cell fate, structure and function. Careful selection of an appropriate ECM is especially critical for co-culture models, because the materials need to support a range of different functions in multiple cell types. Organoid cultures are traditionally maintained in Matrigel, a gel-forming basement membrane material composed of laminin, collagen type IV, entactin and heparan sulfate (Aisenbrey and Murphy, 2020). While Matrigel allows strong organoid growth, its composition is not very well defined, and there is significant batch to batch variability, as we and others have shown (Aisenbrey and Murphy, 2020). Moreover, Matrigel is harvested from transplantable Engelbreth-Holm-Swarm sarcomas propagated in mice, raising animal welfare concerns (Hassell et al., 1980). Because of these multiple limitations of Matrigel as an ECM material, recent studies have analyzed alternative natural and synthetic hydrogels for different tissue culture applications (Aisenbrey and Murphy, 2020; Kaur et al., 2021). In this work, we used a commercially available synthetic hydrogel, VitroGel^®^ ORGANOID 3 (V-ORG-3) (Gebeyehu et al., 2021), which sustained HGO viability and growth while eliminating the batch-to-batch variation of Matrigel.

Importantly, the ECM material also was required to support DC migration and spontaneous DC interactions with the HGO in the GOFlowChip. Although DC can migrate through Matrigel by degrading the matrix with MMPs, as our data and other published studies indicate (Chabot et al., 2006; Adhikary et al., 2012; Cougoule et al., 2018), this process is slow and inefficient, limiting the establishment of physiological cell-cell contacts between DCs and the organoids in our co-cultures. Surprisingly, we determined that diluted Matrigel further inhibited DC migration. This may have occurred due to the often biphasic nature of cell motility, in which high concentrations of adherence molecules prevent cells from releasing adherence to move forward, while low concentrations of adherence molecules slow cells by preventing formation of new attachments (Ranucci and Moghe, 2001; Stroka and Aranda-Espinoza, 2009). Tumor microenvironment MPSs have demonstrated the importance of ECM composition to migration of tumor-infiltrating lymphocytes (Fischer et al., 2017). Notably, migration of immune cells through matrices can be affected by cell activation state. Immature MoDCs have been reported to migrate through both dense gelled collagen and Matrigel or less complex fibrous collagen, but upon activation and maturity, develop impaired migration through dense matrices (Cougoule et al., 2018). We confirmed that our selected ECM material had limited effect on the maturation state of MoDCs. Importantly, DC chemotaxis through V-ORG-3 was significantly increased compared to migration through Matrigel, as shown in our Transwell chemotaxis and GoFlowChip co-culture imaging experiments. Overall, V-ORG-3 supported culture of gastric organoids while maintaining permeability to migrating MoDCs, allowing for enhanced MNP motility and epithelial surveillance within our device.

Immunosurveillance of the gastrointestinal epithelium by MNPs is the “front line” of adaptive immunity within the GI mucosa. MNPs survey the epithelium, taking up luminal antigens including commensal and pathogenic microbiota to establish appropriate gut immune responses (Mann and Li, 2014). By adapting the GOFlowChip for use with MNPs, we have observed these interactions in real-time for up to 20 h. MoDCs in our experimental system were observed migrating towards and surveying the surface of gastric organoids that were embedded V-ORG-3 (Figure 6). Our improved GOFlowChip co-culture system now enables us to analyze the complex interactions between DCs and the gastric epithelium in a controlled microphysiological chip platform. Next step experiments will utilize our GOFlowChip co-culture platform to study mechanisms of DC antigen uptake across the epithelial barrier as a key step in the induction of antigen-specific immunity. While *in vivo* animal models for MNP-epithelial interactions are available (Jia and Edelblum, 2019), our experimental system utilizes human organoids and cells, eliminating confounding species-specific structural and immunological differences.

As drug candidates evaluated using animal models frequently fail when entering human trials, many researchers and industry experts predict that MPSs will become a valuable step in development of novel therapeutics (Low and Tagle, 2017; Tagle, 2019; Peters et al., 2020). Development of gastrointestinal MPSs may have important applications for more efficient drug screening tools, as gastrointestinal toxicities are some of the most common adverse events reported during clinical trials, and they generally involve immune cell activation (Tamaki et al., 2013; Federer et al., 2016; Peters et al., 2020). For example, half of patients undergoing chemotherapy develop toxicity-induced gastrointestinal mucositis. Damage to the epithelial barrier and increased inflammation may lead to a dysregulated microbiome, improper wound healing and further infiltration and epithelial barrier disruption by inflammatory immune cells (Perfetto et al., 2003). Integration of immune components into MPS designed to study these toxicities will strengthen the physiological relevance of these disease models. Of note, MNPs can be a particular hinderance to development of nanoparticle-based drug delivery systems. When patrolling and sampling their environment for potential pathogens or tissue damage, MNPs similarly phagocytose nanoparticles, removing them from their intended delivery site (Yong et al., 2017). The GOFlowChip with co-cultured 3-D organoids and DCs may be a useful pre-clinical model to investigate such potential undesirable interactions.

In conclusion, our improved GOFlowChip co-culture system enables us to analyze the complex interactions between DCs, the gastric epithelium, and the microbiota, broadening our studies of MNP immunosurveillance and its role in gastric inflammation and disease. Our model can be adapted for study of other areas of the gut with relative ease, making our design relevant to multiple tissue types and disease models. Importantly, by identifying an ECM material which supports culture of organoids and motility of immune cells, we have overcome the batch-to-batch variation and hinderance to immune cell motility of Matrigel, a serious obstacle to increasing biological complexity of organoid tissue chip models. The successful integration of immune cells into our gastric organoid tissue chip extends its physiological relevance and applicability, and we anticipate that our findings will inform design optimizations for other immune-cell-organoid culture platforms used in basic and translational biomedical research.

## Data Availability

The raw data supporting the conclusion of this article will be made available by the authors, without undue reservation.

## Supplementary Material

Supplementary Figure S1 Human gastric organoids retain their circular morphology and size in V-ORG-3 for 20 h. Top panels: Representative confocal images of mCherry-expressing HGOs 0 h and 20 h after transfer from Matrigel to V-ORG-3, maximum Z-projection. Bottom panels: bright field images of the same HGOs shown in the top panels. Bar = 200 µm.

Supplementary Video S1 Poor movement of MoDCs (green) when co-cultured with and HGOs (red) embedded in Matrigel. Co-cultures of MoDCs and HGOs on the GoFlowChip were imaged over 20 h using time lapse confocal microscopy. MoDC movement was analyzed using the particle tracking software Imaris^®^. Cell tracks are shown by colored lines. One of two replicate experiments is shown.

Supplementary Video S2 Improved migration of MoDCs (green) towards HGOs (red) embedded in V-ORG-3. Co-cultures of MoDCs and HGOs on the GoFlowChip were imaged over 20 h using time lapse confocal microscopy. MoDC movement was analyzed using the particle tracking software Imaris^®^. Cell tracks are shown by colored lines. One of two replicate experiments is shown.
