# Supplementary figures and images for: A Synthetic Hydrogel, VitroGel® ORGANOID-3, Improves Immune Cell-Epithelial Interactions in a Tissue Chip Co-Culture Model of Human Gastric Organoids and Dendritic Cells
Source: Front Pharmacol. 2021 Sep 6;12:707891. doi: 10.3389/fphar.2021.707891 (PMC8450338; doi:10.3389/fphar.2021.707891)

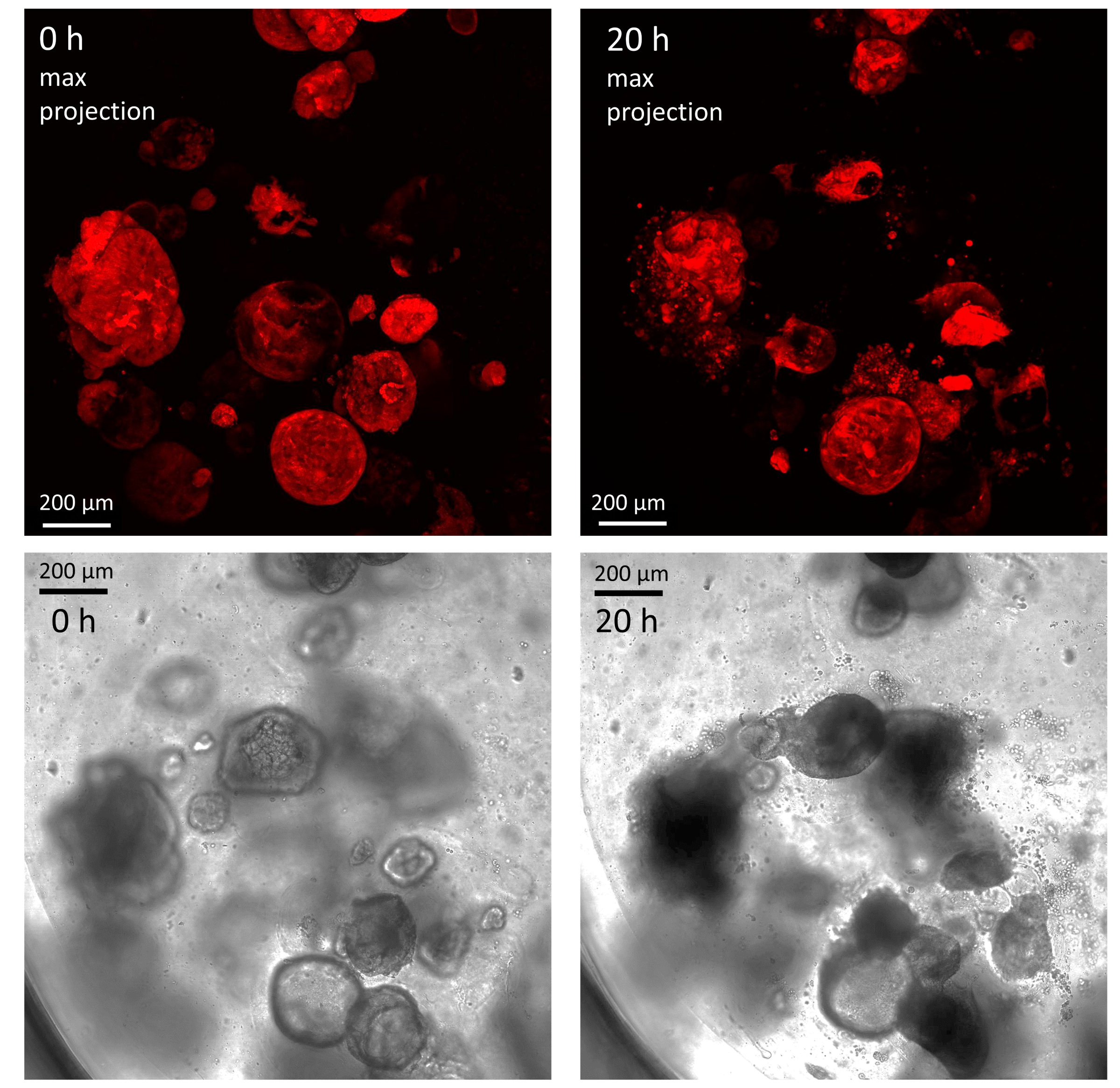

Supplement: Supplementary file 1 [file Image1.TIF]
